# *"Half plate of rice to a male casual sexual partner, full plate belongs to the husband": *Findings from a qualitative study on sexual behaviour in relation to HIV and AIDS in northern Tanzania

**DOI:** 10.1186/1471-2458-11-957

**Published:** 2011-12-28

**Authors:** Joseph R Mwanga, Gerry Mshana, Godfrey Kaatano, John Changalucha

**Affiliations:** 1 National Institute for Medical Research, P.O. Box 1462, Mwanza, Tanzania

**Keywords:** Sexual behaviour, Socio-economic and cultural contexts, Structural drivers of HIV epidemic, AIDS, Tanzania

## Abstract

**Background:**

A thorough understanding of the contexts of sexual behaviour of the people who are vulnerable to HIV infection is an important component in the battle against AIDS epidemic. We conducted a qualitative study to investigate perceptions, attitudes and practices of sexually active people in three districts of northern Tanzania with the view of collecting data to inform the formulation of appropriate complementary interventions against HIV and AIDS in the study communities.

**Methods:**

We conducted 96 semi-structured interviews and 48 focus group discussions with sexually active participants (18-60 years of age) who were selected purposively in two fishing and one non-fishing communities.

**Results:**

The study revealed a number of socio-economic and cultural factors which act as structural drivers of HIV epidemic. Mobility and migration were mentioned to be associated with the risk of HIV acquisition and transmission. Sexual promiscuous behaviour was common in all study communities. ***Chomolea***, (a quick transactional sex) was reported to exist in fishing communities, whereas extramarital sex in the bush was reported in non-fishing community which was predominantly Christian and polygamous. Traditional practices such as ***Kusomboka ***(death cleansing through unprotected sex) was reported to exist. Other risky sexual behaviour and traditional practices together with their socio-economic and cultural contexts are presented in details and discussed. Knowledge of condom was low as some people mistook them for balloons to play with and as decorations for their living rooms. Acute scarcity of condoms in some remote areas such as ***vizingani ***(fishing islands) push some people to make their own condoms locally known as ***kondomu za pepsi ***using polythene bags.

**Conclusions:**

HIV prevention efforts can succeed by addressing sexual behaviour and its socio-economic and cultural contexts. More innovative, interdisciplinary and productive structural approaches to HIV prevention need to be developed in close collaboration with affected communities and be closely related to policy-making and implementation; to go beyond the limited success of traditional behavioural and biomedical interventions to particularly address the underlying social and structural drivers of HIV risk and vulnerability in the study communities.

## Background

An estimated 33.3 million people were living with HIV in the world at the end of 2009 [1]. Sub-Saharan Africa still bears an inordinate share of the global HIV burden with 22.5 million people (more than two-thirds) of the global total infected with HIV; with more women than men living with HIV. Heterosexual transmission accounts for the majority of these infections [1,2]. Tanzania is among the countries affected greatly by HIV and AIDS with 1.3 million people, including adults and children aged less that 18 years, in Tanzania mainland were living with HIV by early 2008 [3], and specific sub-populations in Tanzania among others, fishing communities have disproportionately high HIV burden [4]. As HIV continues to spread and affect the lives of millions of people, discovering ways to prevent the transmission of HIV is of primary concern to health care authorities worldwide and a growing sense of urgency has developed about the imperative need to stop the epidemic.

Sexual behaviour is one of the most significant factors in the spread of HIV as the vast majority of people newly infected with HIV in sub-Saharan Africa are infected during unprotected heterosexual intercourse [1,5,6]. However, most of the studies on sexual behaviour related to HIV transmission do not adequately address contexts which motivate such behaviour [7-9], and more importantly the content and meaning of sexual behaviour have not yet been studied systematically by addressing sexual culture. Public health discourse and campaigns still tend to ignore for instance, notions of sexual pleasure and desire to mention a few, or to focus largely on negative aspects of sex and sexuality. Negative messages about sexuality can undermine, rather than promote, safer sexual behaviour [10].

Many current HIV prevention efforts are failing. Much of the programme and research efforts have been dominated by individual-level behaviour interventions that seek to influence individuals' knowledge, attitudes and behaviour, such as promotion of condom use, treatment of sexually transmitted infections (STIs), sexual health education and recently male circumcision. These interventions are necessary, but are not sufficient to prevent HIV transmission. Despite often high levels of awareness and knowledge of HIV, complex social and cultural norms embedded in the social fabric and the underlying structures, all too often reinforce risk and inhibit behaviour change [11,12]. Furthermore, stand alone behavioural interventions have proved to be ineffective [13], and a combination with structural interventions is being advocated [12]. However, combination HIV prevention requires action simultaneously both on the immediate risks and on the underlying drivers of the local epidemic. These drivers include the physical, social, cultural, organisational, community, economic, legal and policy features of the environment that affect HIV risk behaviour and vulnerability. Stigma and discrimination, entrenched gender inequalities, gender-based violence, human rights violations, mobility and economic power are some of the major structural drivers that hamper HIV prevention efforts and impede progress towards universal access [12].

The main objective of this study was to investigate HIV-related sexual behaviour and its socio-economic and cultural contexts among sexually active people of three districts of northern Tanzania under Sida district development programme namely Bunda, Ukerewe and Serengeti; with the aim of collecting data to understand the socio-cultural environment in which previous interventions were developed and inform the development of new HIV prevention interventions. In the context of this study, Bunda and Ukerewe districts are referred to as fishing communities whereas; Serengeti district is referred to as non-fishing community.

Specifically the study intended: (i) To investigate peoples' perceptions of HIV and AIDS; and socio-economic and cultural factors related to the risk of HIV infection; (ii) To explore sexual behaviour of rural fishing and non-fishing communities in Mwanza and Mara regions; and (iii) To develop appropriate interventions against HIV and AIDS among rural communities of Tanzania. In this study risky sexual behaviour of the study communities in relation to HIV and AIDS is explored in the specific socio-economic and cultural contexts in which it occur and the meaning which are attributed to these practices.

Previous HIV interventions which were implemented in the study areas were: *MEMA kwa Vijana *interventions, a sexual and reproductive health programme for young people in primary schools of four districts of Mwanza region: Kwimba, Misungwi, Sengerema and Geita. The interventions consisted of three components: a teacher-led, peer-assisted primary school health education on AIDS programme, training health workers to encourage youth friendly reproductive health services, youth condom promotion/distribution and community mobilisation [14].

The main objective of sexual and reproductive health for young people in Bunda was to improve the health and livelihoods of young people aged 10-24 years old, with the purpose of promoting responsible sexual behaviour among the people through information, education and communication activities and support for gainful employment. In Serengeti district the programme aimed at improving the reproductive health status of the people with the purpose of strengthening the district capacity to deliver an integrated and comprehensive HIV/AIDS/STIs control programme. Main activities included developing multi-capacity building activities, and the provision and management of grants to implementing partners [AMREF, unpublished report].

## Methods

In 2006 we conducted a qualitative study on HIV and AIDS in three districts of Bunda, Ukerewe and Serengeti in the northern Tanzania. Ukerewe district is an Island on the Lake Victoria and belongs to Mwanza region whereas; Bunda which borders the said lake and Serengeti districts which is located in the hinterland bordering Serengeti National Park, belong to Mara region. Prior to data collection, we visited all study districts in order to introduce the study and make appointments for interviews and discussions. A purposive sampling design was adopted. In each district, study participants were selected from administrative wards, villages and sub-villages (hamlets) with the help of local leaders. Sub-village leaders were informed of the study purpose three days before and in turn they mobilised people to turn out at agreed places where participants were explained the purpose and other procedures of the study. Registers bearing names of members of each household were used to select study participants (sexually active adults 18-60 years of age).

Two people (one male and one female) were selected from each of the 24 sub-villages, making a total sample of 96 people who were interviewed using a semi-structured interview (SSI) schedule in *Kiswahili*, the national language. Four trained graduate research assistants, (two females and two males), were involved in conducting face to face interviews which were noted down manually during the interviews. The interview explored a number of issues apart from socio-demographic characteristics such as perceptions on HIV and AIDS, socio-economic and cultural issues related to sexual behaviour, HIV prevention and AIDS interventions. In the same vein, other people from the same communities were purposively selected from two age-groups (young people between18-24 years of age and adults between 25-60 years of age) to participate in focus group discussions (FGDs).

FGDs were held separately for males and females so as to enhance free and spontaneous discussions. Homogeneity of FGD participants in terms of socio-economic status and educational background was observed [15]. The number of participants in each group ranged from 8 to 12 people. The FGD guide in *Kiswahili *was used to facilitate the discussions. One research assistant assumed the role of moderator (facilitator) and another one was a recorder (notes taker). The FGDs explored a number of issues such as perceptions on HIV and AIDS, risky sexual behaviour and their socio-economic and cultural context, and preventive measures taken against HIV infection. FGDs were both tape-recorded and also noted down manually. A total of 517 people participated in 48 discussion sessions; two sessions in each of 24 sub-villages, which were balanced for gender. This methodological triangulation allowed us to validate data and develop a more in-depth understanding of sexual behaviour of the study population in relation to HIV and AIDS.

Data from FGDs were transcribed verbatim together with reports from SSIs were translated into English. Each transcript was examined line-by-line using NVivo qualitative software (Pty Ltd, Sydney, Australia). Texts were coded using a coding scheme which was prepared beforehand. Emerging theme codes were also added as they appeared. Major theme categories were reviewed numerous times by two social scientists (JRM and GM) to ensure that classification was adequate. Thematic coding allowed the two social scientists to undertake a grounded analysis. This inductive approach was used in order to allow the data to direct the progression of the intended and emergent themes. Data analysis used explicit, systematic and reproducible methods hence, validation and trustworthiness of the findings was established and conclusions drawn were grounded in evidence [16]. In this article we cite actual data in verbatim quotes from interviewees and FGD participants as a prerequisite of findings to be independently and objectively verified [16]. The study informants' age, sex, occupation and settings are provided at the end of each quotation. Furthermore, in this article non-English words in italics are in *Kiswahili *and those in bold italics are of ethnic origin or slang.

Ethical approval for this study was provided by the Tanzanian Medical Research Coordinating Committee. Study objectives, procedures, risks and benefits were explained to participants through an information sheet read/given to them. Thereafter, adequate informed consent of the study participants was sought through signature or thumb print on the consent forms for each tool for data collection. Research results were shared with the study communities and other key stakeholders from the study districts and regions in the form of dissemination workshops and this feedback helped to develop appropriate HIV and AIDS interventions for the study communities (see Discussion section for details).

## Results

### Social and demographic characteristics of interviewees and FGD participants

More than two-thirds of interviewees 68 (71%) were in the age range of 25 and 44 years of age. Slightly more than three quarters of interviewees 74 (77%) were married. The number of female interviewees married as single wives 18 (51%) was almost the same as those married as co-wives 17 (49%). More than two-thirds of interviewees 68 (71%) completed primary education. Peasantry was the main source of livelihood of the interviewees as reported by more than three quarters 73 (76%) followed by fishing-related activities 17 (17%). The majority of interviewees 85 (88%) were Christians. Slightly more than half of Christians 48 (56%) was of Catholic denomination. Regarding FGD participants, more or less the same socio-demographic pattern as that of interviewees emerged. Slightly more than half of participants 297 (57%) were in the age range of 25-44 years of age. Furthermore, slightly more than three quarters of the participants 400 (77%) were married. Majority of participants 372 (72%) completed primary education and another majority 427 (83%) were Christians.

### Mobility and migration as socio-economic and cultural contexts for HIV epidemic

Almost all interviewees and FGD participants were generally in agreement that mobility and migration were associated with the risk for HIV acquisition and transmission. It was reported that fishermen, fish mongers, businessmen/women and all those whose occupation or income generating activities involves travelling from one point to another and leaving behind their spouses; are at more risk of HIV infection as travelling means interacting with many people; hence a possibility of having unprotected sexual intercourse. One participant of adult male FGD provided his own testimony as follows: "*I believe that travelling or migrating contributes to transmitting HIV. For example today I fish dagaa (sardines) here on one island and I have a lover whom I trust and she trusts me. I continue fishing and having sex with her. After some time I move to another island and leave her behind. At another island I get another woman whom I have unprotected sex with and I don't know with whom she was having sex before and whether her former boyfriend or husband had been infected or not; and I continue with the same trend in other fishing posts. This behaviour is common among many of my fellow fishermen." *[FGD participant, 30 years old fisherman from Ukerewe district].

### Risky sexual behaviour and its socio-economic and cultural contexts

#### Sexual promiscuity in pre-and extra-marital sexual relationships

The data from this study open up the much wider issue of the seriousness of the economic crisis facing study communities and its much faceted relationship to the spread of HIV. Material exchange for sex (transactional sex) may be important to sexual relationships and health in certain cultures. In this study, sexual promiscuity was explained to take different forms in both pre-marital and extra-marital sexual relationships which were reported to be common in the study communities. As part of sexual promiscuous behaviour there is a sexual practice among women in the fishing communities locally known as ***chomolea ***(a type of quick paid sex). It was reported that in ***vizingani ***(fishing islands) in Ukerewe district, fishing business is very lucrative and sexual promiscuity in the form of ***chomolea ***is common. One participant of adult male FGD gave the following explanation of the said practice: "***Chomolea ****sex is very dangerous as far as acquisition and transmission of HIV infection is concerned because it is a quick paid sex at any place and time on the island. Only 2,000/ = TShs*. (at the time of study it was approximately 2.00 US $) *is needed per sexual act. It is done under difficult circumstances (in latrines, bushes and sometimes on beds in guest houses on fishing islands); and in many circumstances condom is not used*. " [FGD participant, 40 years old fisherman from Bunda district].

It was further revealed that in ***vizingani ***there are a fairly large number of women who sometimes cook for fishermen (***kaboneke***); but their intention is to sell their bodies. These and others including some married women are the ones who practice ***chomolea***. One participant of adult male FGD remarked: "In ***vizingani ****there are fixed prices to pay for sex; you can have a quick sex with a woman for 2,000/ = TShs*. (approximately 2.00 US $) *and above. During '**mtegeruko**' (pay day for fishermen) **chomolea **sex becomes a bit expensive as it can be exchanged for up to 10,000/ = TShs*. (approximately 10.00 US $). *Now this woman for instance, can have a quick sex with me and after a few minutes can do it with another man. During daytime she can have sex with at least four men and another four during the night. With all those eight men there is a high possibility of getting infected with HIV."*[FGD participant, 29 years old fisherman from Bunda district].

More importantly, in one of the FGDs of young females in Bunda district, ***chomolea ***sex has been variously explained in phrases such as: '*Je unataka nusu wali/nusu sahani ya wali*?' literary means do you want half plate of rice? Referring to casual sex with a male partner which does not involve sleeping overnight, whereas '*Je unataka sahani nzima ya wali*?' (Do you want a full plate of rice?) Referring to having sex with a male casual partner overnight; and phrases such as '*nusu wali kwa hawara, sahani kwa mume' *(meaning-casual sex with a male partner and sleeping overnight with a husband) were very common.

Extra-marital sex in the bush was another form of sexual encounter reported by FGD participants, particularly from non-fishing community. One participant of adult male FGD gave his own testimony as follows: "*It was 1998; there was a married woman whom I fell in love with. One day she told me to wait for her somewhere in the bush. She came with fried chicken and rice. We ate; it was very delicious meal and later on had sex." *[FGD participant, 42 years old male pastoralist from Serengeti district].

### Other forms of risky sexual relationships

#### Wife-inheritance

Inheritance of widows at the death of a husband used to be a common practice among the people in many parts of Tanzania. A woman whose husband died is forced to get married to another man from the family of the deceased husband on the pretext of providing security to dependent survivors as well as preserving the lineage. In this study it was also reported that wife inheritance used to be common among both fishing and non-fishing communities in the past but with the advent of HIV and AIDS; these practices appear to be diminishing. However, there is anecdotal evidence that it is still being practiced, though rarely. One participant of young female FGD admitted as follows:*"Wife inheritance is a good thing as you only have sex within the clan; even if you get pregnant you won't give birth to a thief. It is a good luck and the clan will praise you because you have proved that you are not a prostitute." *[FGD participant, 24 years old female from Serengeti district].

#### Wife-sharing

Wife-sharing in the family in the form of in-law relationships were also reported to exist among some ethnic groups in both fishing and non-fishing communities. One participant of adult female FGD made the following comments:*"The *(ethnic group name withheld) *women are permitted to have sex with brothers-in-laws. The husband leaves the house for his brother to have sex with his wife without any interference. Also a husband can have sex with as many women as he can; and a wife is not allowed to question. This is because jealousy is strictly prohibited in this ethnic group." *[FGD participant, 45 years old female from Serengeti district]. The practice of sharing wife in a clan was also reported in Bunda district. One interviewee remarked: "*Among a small community *(name withheld), *there is a practice of a father of the groom to have sex with his daughter-in-law before his son so as to bless his marriage." *[Interviewee, 20 years old young male from Bunda district].

### Risky traditional practices

#### Death cleansing through unprotected sexual intercourse

Each culture defines what is pure and impure (polluted) and the consequences of purity and pollution differently from every other culture. In case of the pollution of death rituals are performed to re-establish lost purity. The traditional practice known as *kuosha kifo/kusafisha kifo/kuondoa mkosi wa kifo *locally referred to as ***kusomboka ***(death cleansing/removing death jinx) through unprotected sexual intercourse with someone not known to survivors was reported to exist in fishing communities. One interviewee described the said tradition as follows: "*When a husband/wife dies, a widow/widower has to undergo ****kusomboka ****to prevent similar event(s) to recur in future. This involves for instance a widow having unprotected sex with a man not known to her. This function is sanctioned by the clan and a man who is involved in this 'ritual purity' is paid a cow, a goat or money*." [Interviewee, 50 years old male from Ukerewe district].

#### Female to female marriages (nyumba ntobu)

Marriage practices have been and continue to be undergoing major transformation in Africa. The meaning and practice of marriage and its relationship to sexual access, is continually undergoing renegotiation. Many different, socially recognised forms of liaison may operate in any given context for example a traditional practice which interviewees reported to contribute to the spread of HIV known as ***nyumba ntobu ***(literary means female to female marriages) which is common in Serengeti district and particularly among one ethnic group (name withheld). One of the interviewees gave the following explanation of the said tradition: *"A barren woman or a woman who has daughter(s) and therefore in need of a child/son marries another woman who can bear a child for her by another man she chooses for her*." [Interviewee, 45 years old female from Serengeti district].

#### Traditional circumcision practices

Male circumcision (MC) is regarded as one of the oldest surgical procedures in the world and has been practiced by communities in different parts of the world for among others, cultural and religious reasons. Almost all interviewees 94 (98%) from both fishing and non-fishing communities admitted that (MC) is being practiced in their communities mainly among the boys as a rite of passage, to keep them clean and to prevent infections. However, among a certain ethnic group (name withheld) in Serengeti district even young females undergo circumcision by traditional circumcisers for cultural reasons as a rite of passage which sometimes assumes the proportions of female genital mutilations. Participants raised their concern that such traditional practices although have cultural values attached to, if they are carried out in unhygienic conditions (i.e. by sharing un-sterilised surgical instruments such as knives, razor blades etc.), can facilitate transmission of HIV infection.

### Overnight social functions coupled with excessive alcohol consumption

Overnight social functions coupled with excessive alcohol consumption were reported to be other social contexts for HIV infection. Majority of interviewees from fishing communities 80 (83%) reported that some uncontrolled social functions such as discos and wedding ceremonies were characterised by alcohol abuse and unsafe sexual behaviour among quite a number of adults who attend. Majority of interviewees 87 (91%) were in agreement that excessive alcohol consumption was one of the key contributing factors in acquisition and transmission of HIV infection in their communities. One interviewee said: "*Alcoholism is also a factor in transmission of HIV. Those who drink heavily can have hyper libido; they can decide to take women and have unsafe sex." *[Interviewee, 21 years old young male from Ukerewe district].

Furthermore, majority of interviewees 85 (88%) reported such unsafe sexual behaviour among the youth during overnight discos locally known as ***disko vumbi ***(dusty disco), where alcohol is consumed in excess. Both girls and boys may get drunk and fall victim of unplanned and unsafe sex or a girl may even be raped. One interviewee remarked: "*Yes, during ****disko vumbi ****youth go for wanenguaji (stage show girls) particularly during msimu wa pamba (cotton buying season) when youth have money from cotton sales and have sex with them. With stage show girls hutongozi (you do not seduce); you pay money to DJ (Disc jockey) between. 5,000/ = TShs. and 10,000/ = TShs. (approx. between 5 US $ and 10 US $) for a girl and take her for sex." *[Interviewee, 19 years old young male from Ukerewe district]. In the same vein, another interviewee remarked: "*In our village there was one businessman who used to sell stage show girls to earn money." *[Interviewee, 22 years old young male from Bunda district].

### Preventive measures against HIV and AIDS

Concerning how to protect themselves against HIV infection a minority of the interviewees from both fishing and non-fishing communities 38 (40%) painted a bleak picture of condoms. They mentioned that some people have not seen and do not know a condom. One interviewee said: *"I once placed condoms in my pocket and they dropped under the bed; when my wife saw them she thought they were vidonge vya ng'ombe (tablets for cattle)." *[Interviewee, 49 years old male pastoralist from Serengeti district]. In the same vein, another interviewee made the following comments: "*Some women who are given male condoms at the dispensary do not know their use. They give them to children to play with as balloons. Sometimes the balloons made out of condoms are hung in their seating rooms as decorations*." [Interviewee, 24 years old female from Serengeti district].

Another issue which emerged during the FGDs was the non-availability of condoms especially away from district towns and village centres; and in ***vizingani ***or often erratic supplies through channels and distribution points, which were not well known to everybody in the community. They reported that scarcity of condoms push people to engage in unprotected sex. One participant of adult male FGD said: *"When you meet a woman in a difficult environment such as when collecting firewood, fetching water or grazing cattle; and there are no condoms around you end up having unprotected sex with her." *[FGD participant, 37 years old male from Serengeti district].

Apart from being scarce, condoms were also reported to be expensive for some people. FGD participants reported that unavailability of condoms and/or the fact that condoms are expensive (100/ = TShs.- approximately 0.1 US $ cent, at the time of the study) for a pack of three condoms of *salama *brand contribute to forcing people to engage in unprotected sex or even resort to making their own condoms locally referred to as ***kondomu za pepsi ***which are both risky options. One participant of young male FGD described how local condoms are made as follows: "*One takes a plastic (polythene) bag and cuts out a shape of an ordinary condom into two pieces. You see... and then apply a strong glue (e.g. pattex) to hold together the two pieces and leaves a space; a size of a male sexual organ to go through and bend the upper end to make a rim which is made of rubber band. Can't you see that this very risky? It is better you bring free condoms to these people rather than leaving them to make their own just like that." *[FGD participant, 18 years old young male from Bunda district].

## Discussion

### Risky sexual behaviour and other socio-economic and cultural contexts for HIV infection

In this study mobility and migration were found to be among the socio-economic and cultural contexts predisposing mobile population to HIV infection. Another study in Tanzania found the same factors which are associated with occupational group such as fishermen to be important risk factors for HIV transmission [17]. Other studies conducted in Tanzania have shown that numerous aspects of migration-being separated from one's spouse (both the traveller and the one staying behind), the frequency of travel, and the duration of time away from home-impact on risky sexual behaviour [18]. High levels of short and long term mobility and being close to the trading centre were significantly associated with increased HIV incidence [19]. More importantly, a risky sexual behaviour occurred more often in mobile, co-resident men, and in women living apart from their husbands, who infrequently see them, than in men and women who are separated for long periods of time [20].

A study on commercial motorbike-taxi riders, locally known as ***bodabodamen ***an indigenous employment group that is highly mobile but have not been studied before, has gone beyond epidemiological parameters and tried to highlight the social conditions which act as opportunities for HIV infection [21]. However, it should be noted that mobility is not always associated with risky sexual behaviour. One study in Tanzania, for example, found that the ***Maasai ***men do not have sex outside wedlock when they travel to towns on business [22]. Furthermore, it was also revealed that extramarital sexual relationships were not exceptional even in non-fishing community where polygamy and Christianity are widely practiced. This finding is intriguing as polygamy and Christianity are highly incompatible.

Although the extent of transactional sex (exchange of sex for material support including money and gifts) is not known, it is known that there are various forms of transactional sex taking place in Tanzania, ***chomolea ****sex*, which was found to be common in study communities is one of them. However, the line between transactional sex and female sex work is blurred. Many of the women who have had sex in return for gifts, money and the like would not classify themselves as sex workers. One study in a mining town in northwest Tanzania for instance, found many types of women receive payments for sex, distinguished by permanency of residence, age, relationship status, accommodation and income-earning activity, and that such activities were most likely to take place in towns, as a result of economic opportunities available there (in contrast to the poverty surrounding areas), which were often accessed by offering sex in exchange for money or gifts [23]. Despite the argument that motivations for transactional sex, the way it is negotiated, its scale and consequences are still little understood [23,24], transactional sex is likely to increase the risk of HIV and other STIs by encouraging partner change, making relatively affluent men (often higher risk) more desirable, and creating uneven power relationship which makes it difficult to negotiate for condom use [24,25].

The importance of addressing sexual culture in research pertaining to AIDS has been underscored elsewhere [26]. More importantly, behavioural interventions should directly address how embedded transactional sex is in sexual culture [25]. Furthermore, no AIDS prevention programme can afford to ignore the socio-economic aspects of sexual behaviour or operate in isolation from the need for action on poverty and gender inequality. It should be noted that the meaning of sexual behaviour is not as self-evident as it appears. Anthropological accounts start from the premises that sexual behaviour is socially constructed-that is, its content and meaning are determined within social relations. However, the meaning of sex is not necessarily the same across all relationships. For instance, procreative aims may be emphasised fairly exclusively in marital sex but not in non-marital or extra-marital relationships. Knowledge of the extent of the separation of sex from reproductive aims for both sexes will be important both in assessing 'risk' categories and in the formulation of campaigns to promote condom use and safer sex [27].

Among study communities it has also been revealed that there still exist traditional practices such as female to female marriages, death cleansing through unprotected sexual intercourse, wife sharing and inheritance; and traditional male and female circumcision that health interventionists have got to contend with in promoting AIDS control measures. In most cases these traditional practices are sanctioned by clan leaders. A recent study has revealed that traditional circumcision practices for cultural reasons are still common in Tanzania [28]. Furthermore, the practice of inheriting widows and wife sharing in the form of in-laws sexual relationships is also taking place in several districts of Kagera region in Tanzania [The Tanzania Red Cross Society & Danish Red Cross, unpublished report]. Regarding the issue of addressing the afore-mentioned cultural practices, there has been a long-running debate as to whether sexual cultures in sub-Saharan Africa are permissive or characterised by restrictive rules, rituals and self-restraint and the fight against AIDS in Africa is often presented as a fight against 'cultural barriers' [29]. However, we do concur with writers who perceive them as not 'cultural barriers' in the war against AIDS, and therefore should not be seen as promoting the spread of HIV but we should rather try to make such behaviour and practices safer in a way that is culturally acceptable to the people [11,29].

Over-night social functions coupled with excessive alcohol consumption have been found to constitute social contexts for HIV infection in the study communities. Similarly, dusty discos have also been pointed out as social contexts for sexual and reproductive health risks and vulnerability for adolescents in rural Mwanza [30]. In the same vein, alcohol use is a key social behaviour that has been consistently associated with increased risk of HIV acquisition in many settings. In a systematic review and meta-analyses of 20 studies conducted in Africa, alcohol drinkers had 57-70% greater risk of being HIV infected than non-drinkers [31]. There are several factors which may account for the increased risk of HIV among alcohol drinkers. Alcohol has been associated with increased risk of other STIs which are known to facilitate HIV acquisition and transmission [32]. In a review of empirical studies conducted in sub-Saharan Africa, alcohol consumption was consistently related to risky sexual behaviour, including multiple sexual partners and lack of condom use [33]. Thus, alcohol may be associated with high risky sexual behaviour which facilitates HIV acquisition and transmission.

### Preventive measures against HIV and AIDS

Human behaviour is complex; widespread behaviour changes are challenging to achieve; and there are important gaps in our knowledge about the effectiveness of HIV prevention. Yet the research to date clearly documents the impact of numerous behavioural interventions in reducing HIV infection. We also know that in all cases in which national HIV epidemics have reversed, broad-based behaviour changes were central to success [5]. Nevertheless, sexual practices have proven difficult to change. Contemporarily, condom is promoted most often as a device to avoid STIs including HIV. However, condom use often represents a decision to have 'unnatural' or 'undesirable' sex, for example with prostitutes [6]. The use of condoms during sexual intercourse may even become symbolic of suspicion and mistrust. This leads to problems of negotiating condom use among partners who are socially intimate. Therefore, understanding decision-making and power in sexual relationships is crucial to promoting and predicting condom use. However, a recent review of 18 meta-analyses found significant increase in condom use and reductions in unprotected sex [5]. Furthermore, a recent study in Tanzania found that there were significant increases in condom use amongst young women and older men over 15 year period [34]. Nevertheless, the limiting factors with regard to condom use from our study can be summarised as follows: lack of knowledge on how to use them, practical factors such as limited availability of condoms because of inadequate supply, or inaccessibility of stock and financial constraints.

Basing on both the experience from review of the previous interventions designed and implemented in the study communities, study findings and recommendations; and stakeholders views during dissemination workshops; the following is a brief outline of the interventions developed in a form of an intervention manual: Strategies for the provision of action-oriented and community-integrated health education/promotion at all levels; strategies for reaching adolescents; strategic income generating activities for empowerment; developing more gender-sensitive health-financing mechanisms; integration of HIV services with reproductive health services; strategies for reaching orphaned children; provision of medical support/counselling; strategies for addressing harmful cultural practices/behaviour; prevention of HIV infection through syndromic management of STIs; expanding the provision of antiretroviral treatment and condom promotion programme.

## Conclusions

HIV prevention efforts can succeed in the long term by addressing sexual behaviour and its socio-economic and cultural contexts. More importantly, programmes and policies should engage with the ways in which risk and vulnerability are socially embedded. Tackling the social and economic drivers of HIV risk and vulnerability can significantly influence the HIV epidemic if these structural approaches are implemented systematically in combination with behavioural or biomedical interventions. This involves choosing the right mix of HIV prevention actions and tactics to suit the unique epidemic in each country.

## Competing interests

The authors declare that they have no competing interests.

## Authors' contributions

JRM, GM, and JC designed the study. JRM, and GK collected data. JRM, GM, analysed data. JRM drafted the manuscript. All authors contributed to the critical revision of the article and provided final approval for publication.

## Pre-publication history

The pre-publication history for this paper can be accessed here:

http://www.biomedcentral.com/1471-2458/11/957/prepub
